# Incidences of pneumonitis associated with the combination of radiotherapy and immune checkpoint inhibitors in lung cancer: a systematic review and meta-analysis

**DOI:** 10.3389/fonc.2025.1365966

**Published:** 2025-04-17

**Authors:** Jing Li, Lingli Zheng, Chaomin Liu, Wei Liu, Yan Li, Dan Wang, Ting Jiang

**Affiliations:** ^1^ Clinical Medical College, Chengdu Medical College, Chengdu, China; ^2^ Department of Pharmacy, The First Affliated Hospital of Chengdu Medical College, Chengdu, China; ^3^ Department of Oncology, The First Affliated Hospital of Chengdu Medical College, Chengdu, China; ^4^ Department of Oncology, Key Clinical Specialty of Sichuan Province, Chengdu, China

**Keywords:** radiotherapy, immune checkpoint inhibitors, lung cancer, systematic review, meta-analysis

## Abstract

**Objective:**

Recently, the therapeutic effects of the combination of radiotherapy (RT) and immune checkpoint inhibitors (ICIs) on lung cancer (LC) have piqued the interest of the scientific community. Clinical trials have confirmed that RT and ICI therapy exert synergistic therapeutic effects. However, treatment with the RT-ICI combination can lead to the occurrence of pneumonitis, limiting the implementation of the treatment plan, decreasing the tumor control rate, and inducing immunosuppressive pneumonitis and radiation pneumonitis. Therefore, this study performed a systematic review and meta-analysis of pneumonitis prevalence among patients with LC who were treated with a combination of ICIs and chemoradiotherapy (CRT).

**Methods:**

Literature published between January 1, 2010, and October 15, 2023, were searched in the PubMed, Cochrane Library, Embase, and Web of Science databases by two authors. The primary outcomes analyzed in this study were the incidence rates of all-grade pneumonitis and ≥ grade 3 pneumonitis.

**Results:**

This study analyzed 53 studies involving 4226 patients. The pooled incidence rates of all-grade and ≥ grade 3 pneumonitis were 36.0% (95% confidence interval (CI): 30.0–41.0) and 3.0% (95% CI: 2.0–4.0), respectively. The all-grade pneumonitis incidence rates in Asian patients (51%; 95% CI: 38%–63%) were higher than those in non-Asian patients (26%; 95% CI: 22%–31%). Conventional RT was associated with higher rates of all-grade pneumonitis than stereotactic body radiation therapy (SBRT) (37%; 95% CI: 31%–42% vs. 26.0%; 95% CI: 20%–33%). Additionally, sequential immunotherapy was associated with higher rates of all-grade pneumonitis than concurrent immunotherapy ((38%; 95% CI: 31%–45% vs. 25.0%; 95% CI: 20%–30%)). Furthermore, anti-PD1 therapy was associated with higher rates of all-grade pneumonitis than PD-L1 therapy (40%; 95% CI: 32%–47% vs. 20.0%; 95% CI: 16%–24%). Similar incidence rates of ≥ grade 3 pneumonitis were reported in all included studies.

**Conclusion:**

This study suggests that the combination of ICIs and RT/CRT is a safe and feasible therapeutic regimen for patients with LC. However, these findings are based on observational studies and are associated with significant heterogeneity. Hence, large prospective studies are needed to validate the findings of this meta-analysis.

**Systematic Review Registration:**

https://www.crd.york.ac.uk/PROSPERO/#myprospero, identifier CRD42023485613.

## 1 Introduction

Globally, lung cancer (LC), which has the highest mortality rate among all cancers, is the second most common malignant tumor (1, 2). At diagnosis, approximately 50% of patients with LC exhibit distant metastasis. Thus, the overall prognosis of patients with LC is poor (3, 4). Radiotherapy (RT), a traditional therapeutic regimen for non-small cell lung carcinoma (NSCLC), exerts cytotoxic effects on tumor cells and activates the host immune system, suppressing tumor growth and improving prognosis. Research has demonstrated a strong correlation between the incidence of radiation pneumonitis (RP) and both the dosage and methodology of radiotherapy. For example, a specific study examining patients with non–small-cell lung cancer undergoing moderately fractionated radiotherapy reported RP incidence rates of 25.4% and 17.9%, respectively (5). In a separate study examining patients with non–small-cell lung cancer undergoing treatment with volumetric modulated arc therapy (VMAT), the incidence of radiation pneumonitis (RP) reached up to 76%, with 22% of these cases classified as grade 2 or higher (6). Furthermore, research has emphasized that the application of various radiotherapy techniques and dosimetric parameters, including V5, V20, and mean lung dose (MLD), can substantially impact the incidence rate of radiation pneumonitis (RP) (7).

Recently,

Immune checkpoint inhibitors (ICIs) have made significant advancements in the treatment of lung cancer and have been established as a standard therapeutic approach for all stages of non-small-cell lung cancer (NSCLC) and small-cell lung cancer (SCLC). In early-stage and locally advanced lung cancer, the integration of immune checkpoint inhibitors (ICIs) with chemotherapy as a neoadjuvant treatment markedly increases the pathological response rate, extends event-free survival (EFS), and enhances patient prognosis. In the context of advanced lung cancer, the utilization of immune checkpoint inhibitors (ICIs) has become increasingly widespread, integrating into diverse therapeutic regimens including monotherapy, combination with chemotherapy, and dual-immunotherapy strategies. These approaches have markedly enhanced survival rates and quality of life for patients with advanced non-small cell lung cancer (NSCLC) and small cell lung cancer (SCLC). Moreover, immune checkpoint inhibitors (ICIs) have shown a certain level of efficacy in the treatment of lung cancer in later-line settings, providing novel therapeutic options for patients experiencing recurrence or refractory disease. Immune-related adverse events (irAEs), including immune-mediated pneumonitis, are commonly observed side effects in the treatment of cancer patients with immune checkpoint inhibitors (ICIs). Research indicates that the incidence rate of immune-mediated pneumonitis is 12.5% (8). The therapeutic effects of the combination of RT and ICI on LC have piqued the interest of the scientific community. Clinical trials have confirmed that RT and ICI therapy exert synergistic therapeutic effects in cancer. However, the treatment combination of RT and ICI therapy is associated with the occurrence of pneumonitis, limiting the implementation of the treatment plan, decreasing tumor control rate, and promoting the occurrence of immunosuppressive pneumonitis and radiation pneumonitis (RP) (9). Previous studies have demonstrated that RT and immunotherapy may exert synergistic effects through the following mechanisms (10): (a) Exposure of tumor antigen and activation of antigen-presenting cells: RT induces immunogenic cell stress or immunogenic cell death in cancer cells, exposing calreticulin on their plasma membrane and releasing adenosine triphosphate (ATP) and HMGB1. Calreticulin, ATP, and HMGB1 bind to CD91, P2RX7, and TLR4, respectively, facilitating dendritic cell (DC) recruitment into the tumor bed (by ATP), the phagocytosis of tumor antigens by DCs (enhanced by CRT), and an optimal antigen presentation to T cells (stimulated by calreticulin and HMGB1). (b) T-cell priming and activation in lymph nodes: DCs present tumor antigen peptides to T cells. CD40L on the DC surface binds to CD40 of CD4^+^ T cells, activating CD8^+^ T cells. (c) Effector T cells leave the lymph nodes and enter the tumor area and can home to both irradiated and unirradiated tumor deposits and may cause distant tumor regression. In the PACIFIC trial, ICI therapy was initiated within 1–42 days after chest RT (54–66 Gy). The incidence of grade 3 pneumonia in patients treated with ICI was higher than that in patients not treated with ICI (3.4% vs. 2.6%) (11). Zhou Q et al. initiated ICI therapy within 1–42 days after chest RT (54–66 Gy) and reported that the incidence of grade 3 pneumonia was 16% in both ICI and non-ICI groups. However, the incidence of ≥ grade 3 pneumonia in the ICI group was higher than that in the non-ICI group (3% vs. 1%) (12). Jang JY et al. initiated ICI therapy after chest RT (46.0–73.0 Gy) and demonstrated that the incidence of ≥ grade 3 pneumonia was not significantly different between the ICI and non-ICI groups (5.9% vs, 5.4%) (13). Thus, inconsistent pneumonia incidence rates have been reported for the ICI-RT combination. Additionally, these studies involved small sample sizes. The application of RT-immunotherapy combination in patients with unresectable LC is dependent on several factors, such as patient selection, RT dosage and fractionation, and the timing of immunotherapy intervention. Therefore, this study performed a systematic review and meta-analysis of pneumonitis incidences among patients with LC who were treated with the combination of ICI therapy and chemoradiotherapy (CRT).

## 2 Methods

### 2.1 Literature search

This systematic review and meta-analysis followed the guidelines of the Preferred Reporting Items for Systematic Review and Meta-analysis (14) and was registered with PROSPERO (CRD42023485613). Ethical approval was not required for this study as the data were obtained from previously published sources. Literature published between January 1, 2010, and October 15, 2023, was independently searched in the PubMed, Cochrane Library, Embase, and Web of Science databases by two authors. The following keywords were used: (‘Pulmonary Neoplasms’ or ‘Lung Cancer’ or ‘Pulmonary Cancer’ or ‘carcinoma’ or ‘Cancer of the Lung’ or ‘Pulmonary Neoplasm’ or ‘Cancer of Lung,’ ‘non-small-cell lung,’ or ‘non-small cell lung’ or ‘NSCLC’ or ‘Small Cell Lung Cancer ‘or ‘Oat Cell Lung Cancer’ or ‘Small Cell Cancer Of The Lung’ or ‘Carcinoma, Small Cell Lung’ or ‘Oat Cell Carcinoma of Lung’) and (‘radiotherapy’ or ‘radiation treatment’ or ‘radiation therapy’) and (‘PD-L1 ‘or ‘PD-1’ or ‘immune checkpoint inhibitor’ or ‘immunotherapy’).

### 2.2 Inclusion and exclusion criteria

The inclusion criteria were as follows: 1) Original studies that included participants with histologically confirmed LC and were treated with the combination of CRT/RT and ICIs (sequentially or concurrently); 2) prospective clinical trials and prospective or retrospective observational studies; 3) studies in which the outcomes included treatment safety; 4) studies published in the English language. The exclusion criteria were as follows: 1) Conference abstracts, case reports, comments, reviews, animal studies, and mechanistic studies and reviews; 2) studies not reporting data on pneumonitis; 3) multiple studies published using the same dataset; 4) comparative studies that had CRT-alone arms. 5) studies that did not have sufficient data (missing clinical safety data).

### 2.3 Data extraction

The data, including first author name, publication year, country, number of cases, type of study, RT type and dose, follow-up time, treatment regimens (ICIs, patterns of combination of ICIs and CRT/RT), incidence rates of all-grade and ≥ grade 3 pneumonitis, were independently extracted by two independent investigators.

### 2.4 Quality assessment

Two researchers independently assessed the quality of the literature. The included studies were evaluated based on the Methodological Index for Non-randomized Studies (MINORS). Disagreements were resolved by arriving at a consensus after discussing with the entire team.

### 2.5 Statistical analysis

The primary outcomes of this study were all-grade and ≥ grade 3 pneumonitis incidence rates. The random effect model was used for statistical analysis, which was performed using the software Stata v. 14.0 (StataCorp, TX, USA). The pooled rates of pneumonitis with the corresponding 95% confidence intervals (CIs) were calculated using the inverse variance method. Heterogeneity was assessed using the Cochrane χ^2^ test and the I^2^ statistic. The I^2^ values of 0%, 25%, 50%, and 75% represented no, low, moderate, and high heterogeneity, respectively. Subgroup and sensitivity analyses were performed to explore study heterogeneity and to determine the impact of each study. The following subgroup analyses were performed: study type (prospective studies/retrospective studies), types of ICIs (anti-PD1/anti-PDL1), RT method, combination mode of RT and immunotherapy (sequential/concurrent), and population type (Asian population/non-Asian population). The publication bias was estimated using Egger’s test.

## 3 Results

### 3.1 Literature search

**Figure 1:**
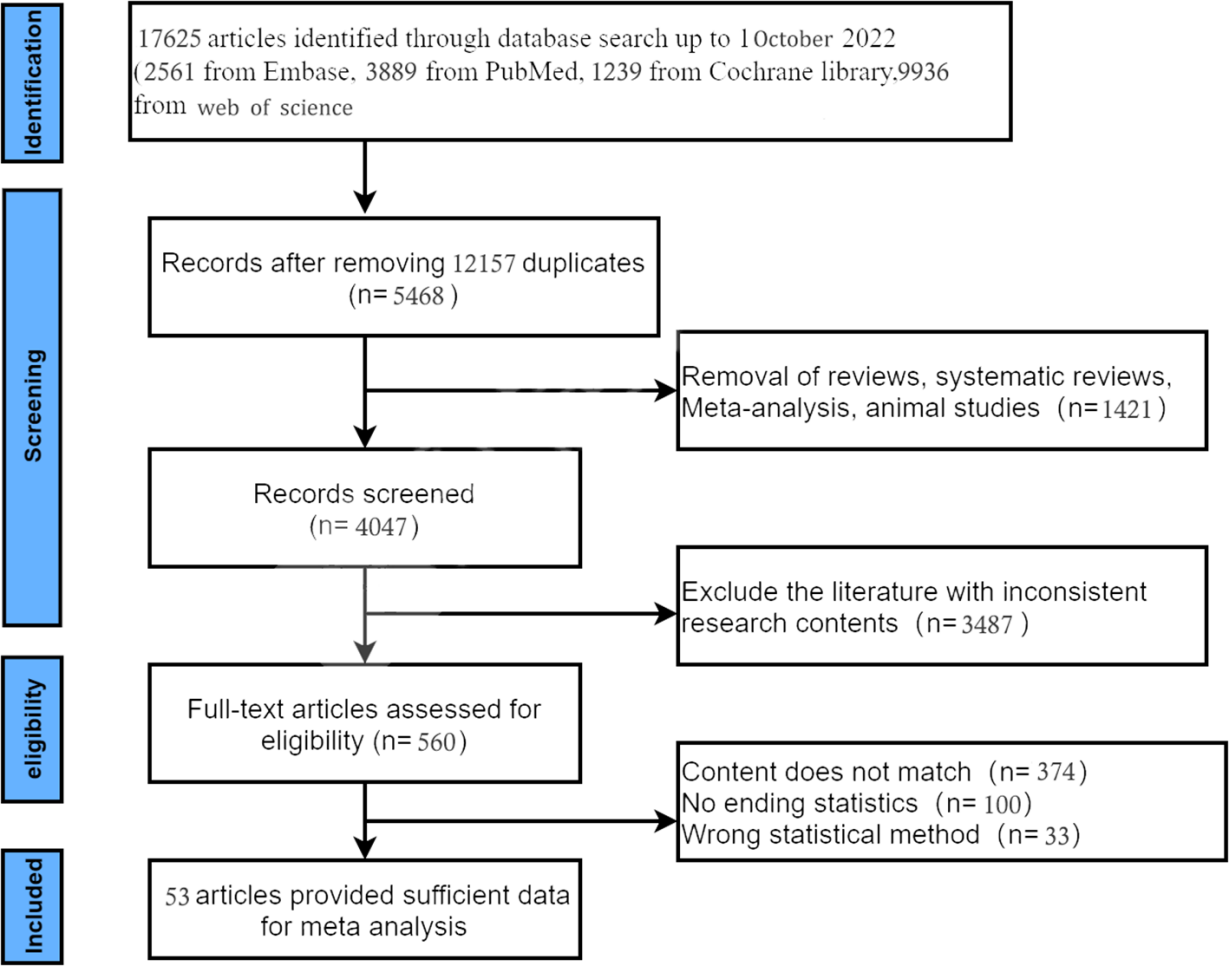
Flowchart of literature search and study screening.

An initial database search yielded 17,625 records. After removing duplicates, 5468 records were obtained. Of these 5468 records, 4704 were excluded based on titles and abstract review. The full text of the remaining 560 articles was retrieved. Finally, 53 studies (11–13, 15–64) involving 4226 patients were included in the analysis. The study selection process and exclusion criteria are shown in **Figure 1**.

The included studies comprised 23 prospective clinical trials and 30 retrospective clinical trials. Most patients in the 53 studies underwent anti-PD1/PD-L1 monotherapy. Patients in three studies underwent anti-CTLA4 therapy. All 53 studies were clinical trials. The methodological quality of these studies was determined using the MINORS scale. The evaluation criteria encompassed the following eight elements, assigning a maximum of 16 points: the inclusion of consecutive patients; an articulated research objective; endpoints that are related to the study aim; prospective data collection; an unbiased assessment of endpoints; a dropout rate of < 5%; a follow-up duration commensurate with the primary endpoint; the prospective determination of the sample size. The scoring system for each criterion was as follows: 0 points if not mentioned in the literature, 1 point if mentioned but with inadequate information or data, and 2 points if mentioned with comprehensive information or data. As shown in **Table 1**, the MINORS scores for these trials varied from 7 to 10 points. Heterogeneity was significant among studies, especially for all-grade (I^2^ = 96.1%) and ≥ grade 3 pneumonitis (I^2^ = 0%). Publication bias was assessed using Egger’s test. Heterogeneity was significant for all-grade pneumonitis (P < 0.05), but not for ≥ grade 3 pneumonitis (p >0.5). The main characteristics of the studies are presented in **Table 1**.

**Table 1:** The main characteristics of the studies.

Author	Year	Nation	N	Study Type	RT Dose/Fraction	Combination Mode	ICI Type	All-Grade Pneumonitis	≥ Grade 3 Pneumonitis	MINORS Score
Araki (30)	2022	Japan	76	Retrospective	50–66 Gy	Sequential	Durvalumab (PDL1)	85.5%	NR	8
Tanaka (20)	2022	Japan	51	Prospective	60 Gy	Sequential	Durvalumab (PDL1)	78.4%	2.0%	9
Abe (44)	2021	Japan	28	Retrospective	60 Gy	Sequential	Durvalumab (PDL1)	60.7%	5.0%	8
Lau (37)	2021	Canada	82	Retrospective	60 Gy	Sequential	Durvalumab (PDL1)	13.0%	2.0%	7
Denault (28)	2022	Canada	150	Retrospective	58–62 Gy	Sequential	Durvalumab (PDL1)	10.0%	3.3%	8
Denault (28)	2022	Canada	52	Retrospective	58–62 Gy	Sequential	Durvalumab (PDL1)	11.5%	5.8%	9
Faehling (55)	2020	Germany	126	Retrospective	65 Gy	Sequential	Durvalumab (PDL1)	15.1%	8.7%	7
Diamond (17)	2023	USA	62	Retrospective	16 Gy	Sequential	Durvalumab (PDL1)	43.5%	9.7%	10
Lin (50)	2019	USA	10	Prospective	60–66 Gy	Sequential	Atezolizumab (PDL1)	30.0%	0.0%	7
Lin (50)	2019	USA	30	Prospective	60–66 Gy	concurrent	Atezolizumab (PDL1)	20.0%	3.0%	7
Herbst (26)	2022	USA	66	Prospective	60 Gy	Sequential	Durvalumab (PDL1)	16.7%	0.0%	8
Käsmann (16)	2023	Germany	11	Prospective	60–66 Gy	Concurrent	Nivolumab (PD1)	64.0%	18.0%	8
Käsmann (16)	2023	Germany	28	Prospective	60–66 Gy	Sequential	Durvalumab (PDL1)	93.0%	14.0%	9
Nishimura (23)	2021	Japan	82	Retrospective	60/64 Gy	Sequential	Durvalumab (PDL1)	74.3%	4.9%	9
Offin (48)	2020	USA	62	Retrospective	54–66 Gy	Sequential	Durvalumab (PDL1)	19.4%	1.6%	7
Oshiro (36)	2021	Japan	91	Prospective	45–66 Gy	Sequential	Durvalumab (PDL1)	88.0%	12.1%	8
Park (15)	2023	Korea	157	Retrospective	54–66 Gy	Sequential	Durvalumab (PDL1)	36.3%	NA	7
Raez (22)	2022	USA	125	Retrospective	54–66 Gy	Sequential	Durvalumab (PDL1)	44.8%	5.6%	8
Saad (21)	2022	Israel	71	Retrospective	56–66 Gy	Sequential	Durvalumab (PDL1)	49.3%	5.6%	8
Saito (34)	2020	Japan	36	Retrospective	60 Gy	Sequential	Durvalumab (PDL1)	75.0%	8.3%	9
Shintani (33)	2021	Japan	146	Retrospective	60–66 Gy	Sequential	Durvalumab (PDL1)	35.0%	5.0%	10
Taugner (31)	2021	Germany	26	Prospective	≥ 60 Gy	Sequential	Durvalumab (PDL1)	NA	15.0%	8
Lu (24)	2022	China	196	Retrospective	NR	Sequential	PD1 + PDL1	55.1%	4.1%	8
Yamamoto (18)	2022	Japan	68	Retrospective	60–66 Gy	Sequential	Durvalumab (PDL1)	33.8%	10.3%	8
Zhang (45)	2020	China	20	Prospective	50–60.2 Gy	Sequential	Durvalumab (PDL1)	80.0%	0.0%	8
Amino (60)	2020	Japan	20	Retrospective	66–74 Gy	Sequential	Pemb (PD-1) (5%) Nivo (PD-1) (95%)	5.0%	0.0%	9
Antonia (11)	2017	USA	709	Prospective	54–66 Gy	Sequential	Durvalumab (PDL1)	33.9%	3.4%	8
Aredo (43)	2021	USA	13	Retrospective	44–69.6 Gy	Sequential	Durvalumab (PD-L1)	23.1%	7.7%	8
Barrón (59)	2020	USA	40	Retrospective	< 60 Gy (52.5%) ≥ 60 Gy (47.5%)	Sequential	Pemb (PD-1) or Nivo (PD-1)	40.0%	10.0%	8
Bestvina (29)	2021	USA	37	Prospective	SBRT 30–50 Gy	Concurrent/Sequential	Pemb (PD-1) Nivo (PD-1)	24.3%	18.9%	9
Bruni (42)	2021	Italy	155	Retrospective	< 60 Gy (7.7%) 60–66 Gy (82.0%) > 66 Gy (10.3%)	Concurrent (58.7%) Sequential (41.3%)	Durvalumab (PD-L1)	17.4%	2.6%	9
Casey (8)	2023	USA	19	Prospective	60 Gy	Concurrent	Ipilimumab (CTLA4)	42.0%	26.0%	8
Chen (41)	2020	USA	33	Retrospective	SBRT (50 or 60 Gy)	Concurrent/Sequential	CTLA-4 + PD-1/PDL-1	18.2%	12.1%	7
Chu (58)	2020	China	29	Retrospective	66–70 Gy	Sequential	Durvalumab (PD-L1)	17.2%	6.9%	7
Eichkorn (56)	2020	Germany	442	Retrospective	60 Gy	Sequential	Durvalumab (PDL1)	15.0%	2.3%	7
Finn (40)	2020	UK	475	Prospective	< 60 Gy (8%) 60–66 Gy (86%) > 66 Gy (6%)	Sequential	Durvalumab (PD-L1)	33.9%	3.6%	10
Garassino (27)	2022	Italy	117	Prospective	60 Gy (10%)	Sequential	Durvalumab (PDL1)	18.8%	1.7%	8
Greg A (57)	2020	USA	93	Prospective	59.4–66.6 Gy	Sequential	Pembrolizumab (PD1)	16.2%	5.4%	9
Hassanzadeh (54)	2020	USA	34	Retrospective	60 Gy	Sequential	Durvalumab (PD-L1)	26.5%	5.9%	8
Inoue (53)	2020	Japan	30	Retrospective	60 or 64 Gy	Sequential	Durvalumab (PD-L1)	73.3%	0.0%	8
Jabbou (52)	2020	Canada	21	Prospective	60 Gy	Concurrent	Pemb (PD-1)	33.0%	10.0%	9
Jang (13)	2021	Korea	51	Retrospective	46.0–73.0 Gy	Sequential	Durvalumab (PDL1)/Atezolizumab (PDL1)/Pembrolizumab (PD1)/Nivolumab (PD1)	52.9%	5.9%	9
Jung (51)	2020	Korea	21	Retrospective	54–66 Gy	Sequential	Durvalumab (PD-L1)	81.0%	14.3%	9
Landman (38)	2021	Israel	39	Retrospective	69.9 Gy	Sequential	Durvalumab (PD-L1)	15.4%	3.0%	10
LeClair (25)	2022	USA	83	Retrospective	< 60 Gy (4%) 60 Gy (48%) > 60 Gy (34%) NR (16%)	Sequential	Durvalumab (PD-L1)	25.3%	7.2%	10
Lin (50)	2020	USA	40	Prospective	60–66 Gy	Sequential (25%)/ Concurrent (75%)	Atezolizumab (PD-L1)	25.0%	2.5%	7
Miura (49)	2020	Japan	41	Retrospective	60 Gy	Sequential	Durvalumab (PD-L1)	61.0%	2.4%	8
Peters (35)	2021	Switzerland	79	Prospective	66 Gy	Concurrent/Sequential	Nivo (PD-1)	16.4%	11.7%	8
Zhou (12)	2022	China	381	Prospective	54–66 Gy	Sequential	Sugemalimab (PDL1)	19.0%	3.0%	9
Salma (39)	2021	USA	112	Prospective	60 Gy	Concurrent	Pembrolizumab (PD1)	19.6%	6.3%	9
Salma (39)	2021	USA	102	Prospective	60 Gy	Concurrent	Pembrolizumab (PD1)	18.6%	4.9%	9
Shaverdian (64)	2017	USA	24	Prospective	NR	Sequential	Pemb (PD-1)	8.0%	4.0%	8
Shukla (32)	2021	USA	92	Prospective	59.40–66.6 Gy	Sequential	Pemb (PD-1)	18.5%	5.4%	8
Tamiya (63)	2017	Japan	50	Retrospective	NR	Sequential	Nivo (PD-1)	22.0%	NA	9
Tian (47)	2019	USA	117	Prospective	SBRT	Concurrent	CTLA-4 + PD-1/PDL-1	33.9%	10.7%	10
Voong (62)	2019	USA	100	Retrospective	60 Gy	Concurrent	PD1 + PDL1	36.0%	21.0%	9
Wass (19)	2022	Austria	36	Prospective	66 Gy	Sequential	Durvalumab (PDL1)	27.8%	2.1%	9
Welsh (46)	2020	USA	80	Prospective	SBRT 50 Gy	Concurrent	Pemb (PD-1)	25.0%	1.3%	9
Yamaguchi (61)	2019	Japan	40	Retrospective	50–60 Gy (60%) 30–40 Gy (40%)	Sequential	Nivo (PD-1)	20.0%	NA	9

RT, radiation therapy; ICI, immune checkpoint inhibitor; SBRT, stereotactic body radiation therapy; MINORS, Methodological Index for Non-randomized Studies; NA, Not Available.

### 3.2 Results of meta-analysis

#### 3.2.1 Pooled analysis of pneumonitis

**Figure 2:**
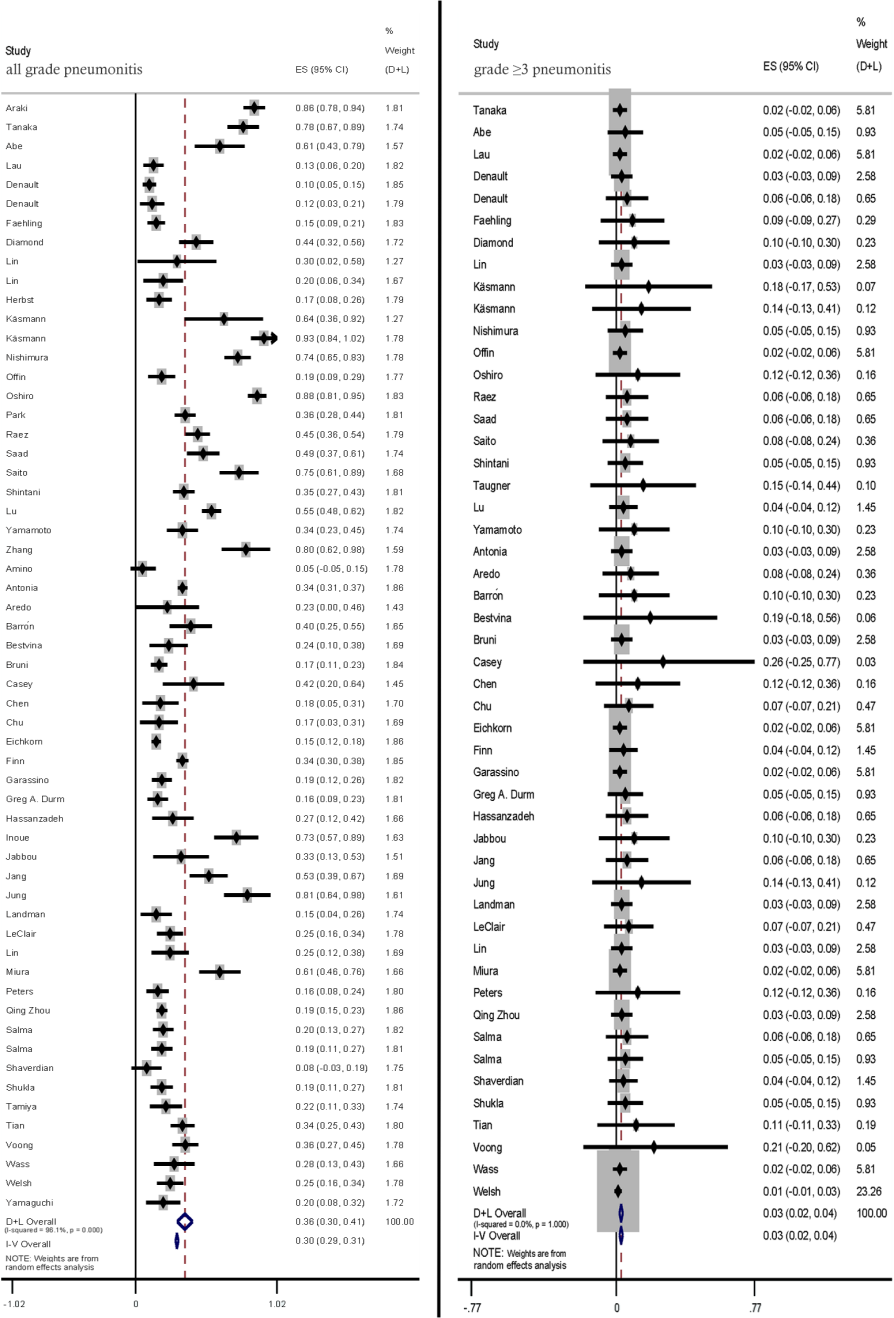
Pooled analysis of all-grade and ≥ grade 3 pneumonitis incidence rates.

Most pneumonitis cases were graded according to the Common Toxicity Criteria for Adverse Events criteria. The pooled incidence rates of all-grade and ≥ grade 3 pneumonitis in patients with LC treated with CRT-ICI combination were 36.0% (95% CI: 30.0–41.0) and 3.0% (95% CI: 2.0–4.0), respectively (**Figure 2**).

#### 3.2.2 Prospective studies and retrospective studies

**Figure 3:**
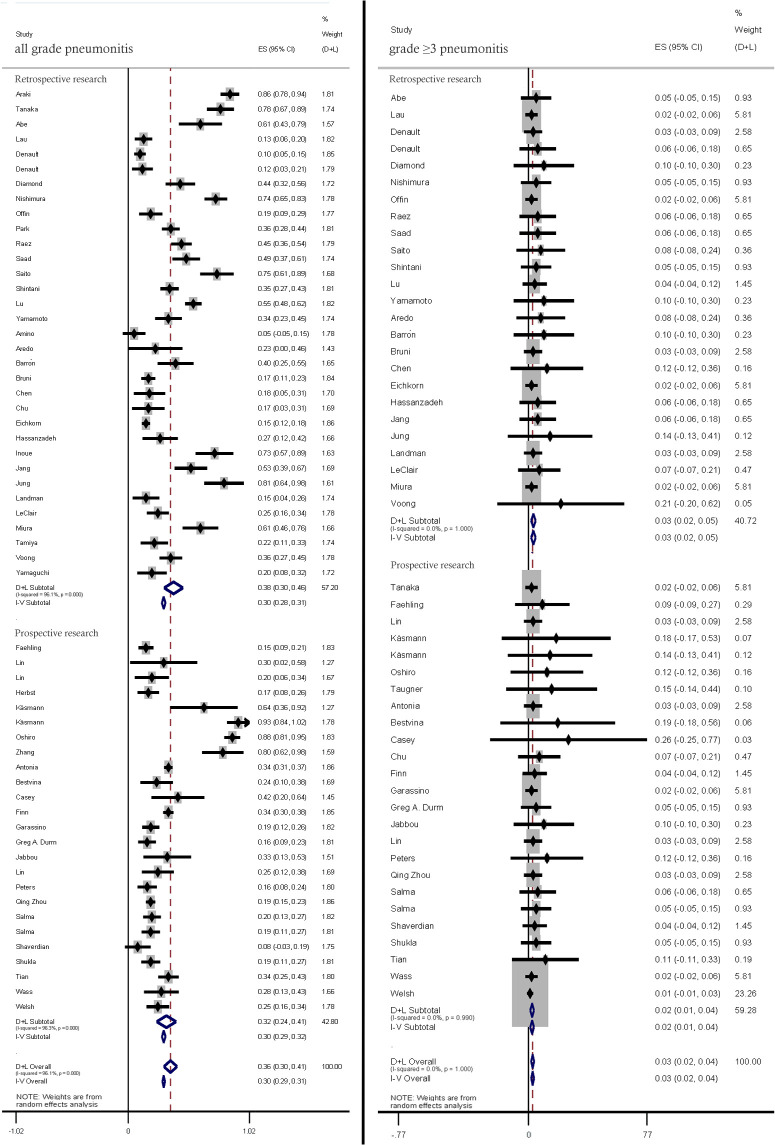
Subgroup analysis of all-grade and ≥ grade 3 pneumonitis incidence rates in prospective studies and retrospective studies.

The incidence rates of all-grade and ≥ grade 3 pneumonitis in prospective studies and retrospective studies were (32%; 95% CI: 24%–41% vs. 38.0%; 95% CI: 30%–46%) pneumonitis (2%; 95% CI: 1%–4% vs. 3%, 95% CI: 2%–5%), respectively. (**Figure 3**).

#### 3.2.3 Conventional RT and stereotactic body RT

**Figure 4:**
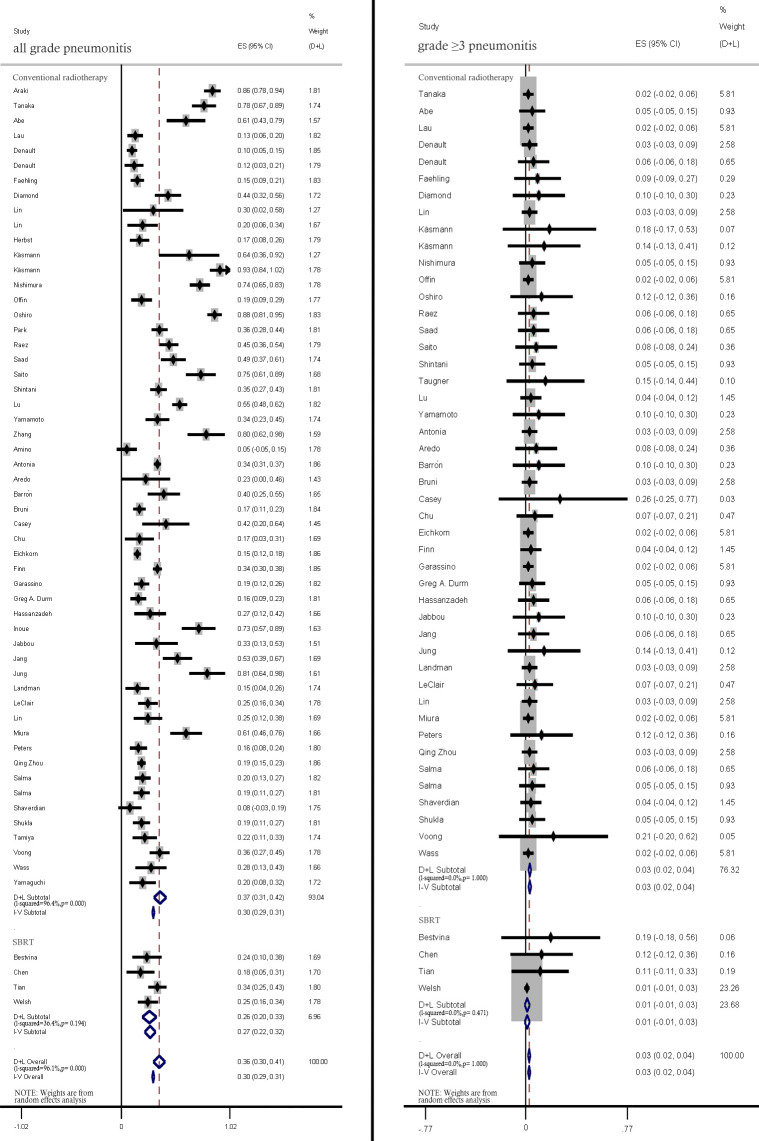
Subgroup analysis of all-grade and ≥ grade 3 pneumonitis incidence rates among patients treated with conventional radiotherapy and stereotactic body radiation therapy (SBRT).

The pneumonitis rates in studies using and SBRT were comparatively analyzed. Compared with those in patients treated with conventional RT, the incidence rates of all-grade (37%; 95% CI: 31%–42% vs. 26.0%; 95% CI: 20%–33%) and ≥ grade 3 pneumonitis (3%, 95% CI: 2%–4% vs. 1%, 95% CI: −1%–3%) were lower in patients with treated with SBRT (**Figure 4**).

#### 3.2.4 Concurrent and sequential immunotherapy

**Figure 5:**
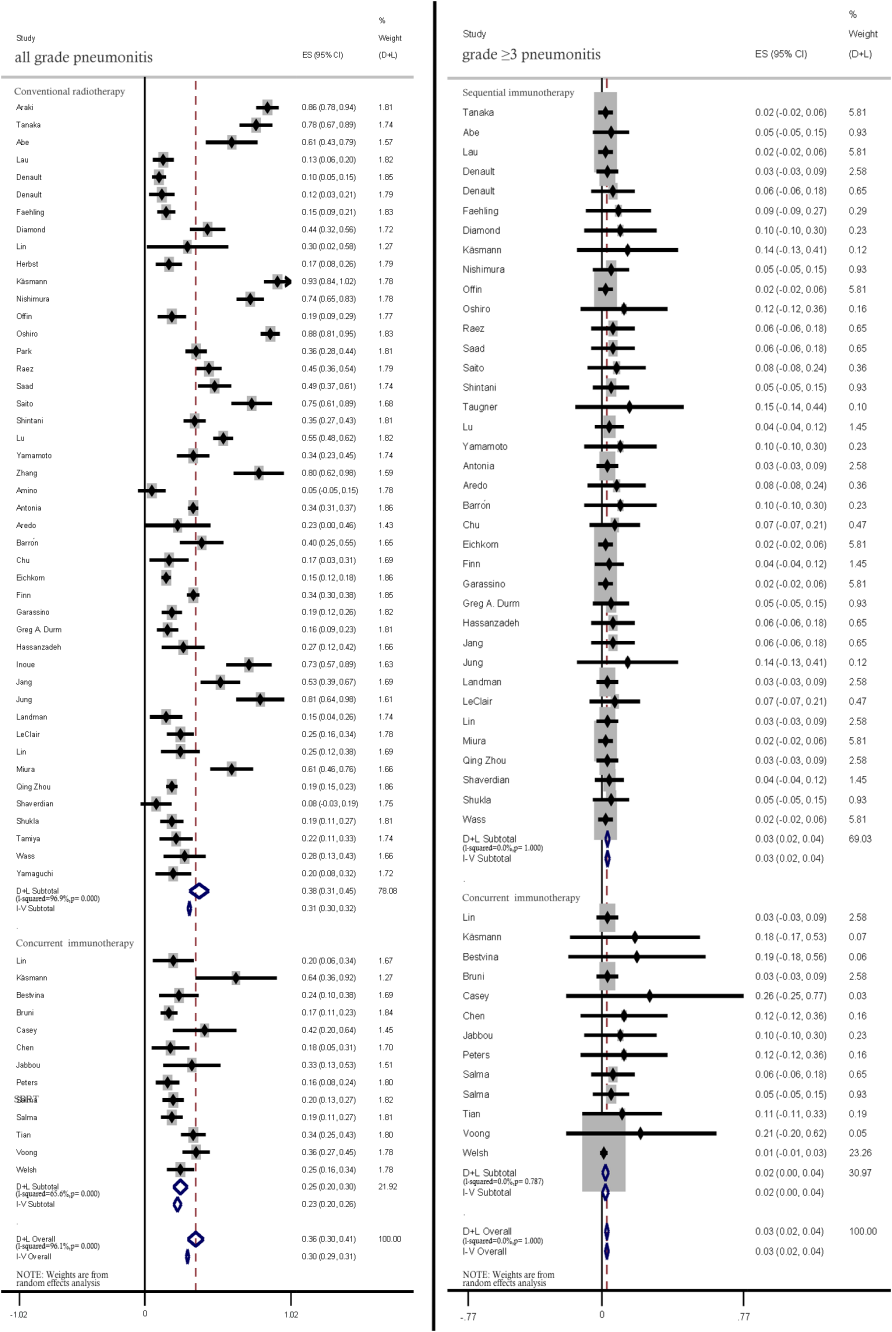
Subgroup analysis of all-grade and ≥ grade 3 pneumonitis incidence rates among patients treated with concurrent and sequential immunotherapy.

Compared with those in patients concurrently treated with CRT and ICIs, the incidence rates of all-grade (38%, 95% CI: 31%–45% vs. 25.0%, 95% CI: 20%–30%) and ≥ grade 3 pneumonitis (3%, 95% CI: 2%–4% vs. 2%, 95% CI: 0%–4%) were lower in patients sequentially treated with CRT and ICIs (**Figure 5**).

#### 3.2.5 Anti-PD-1 and anti-PD-L1 therapies

**Figure 6:**
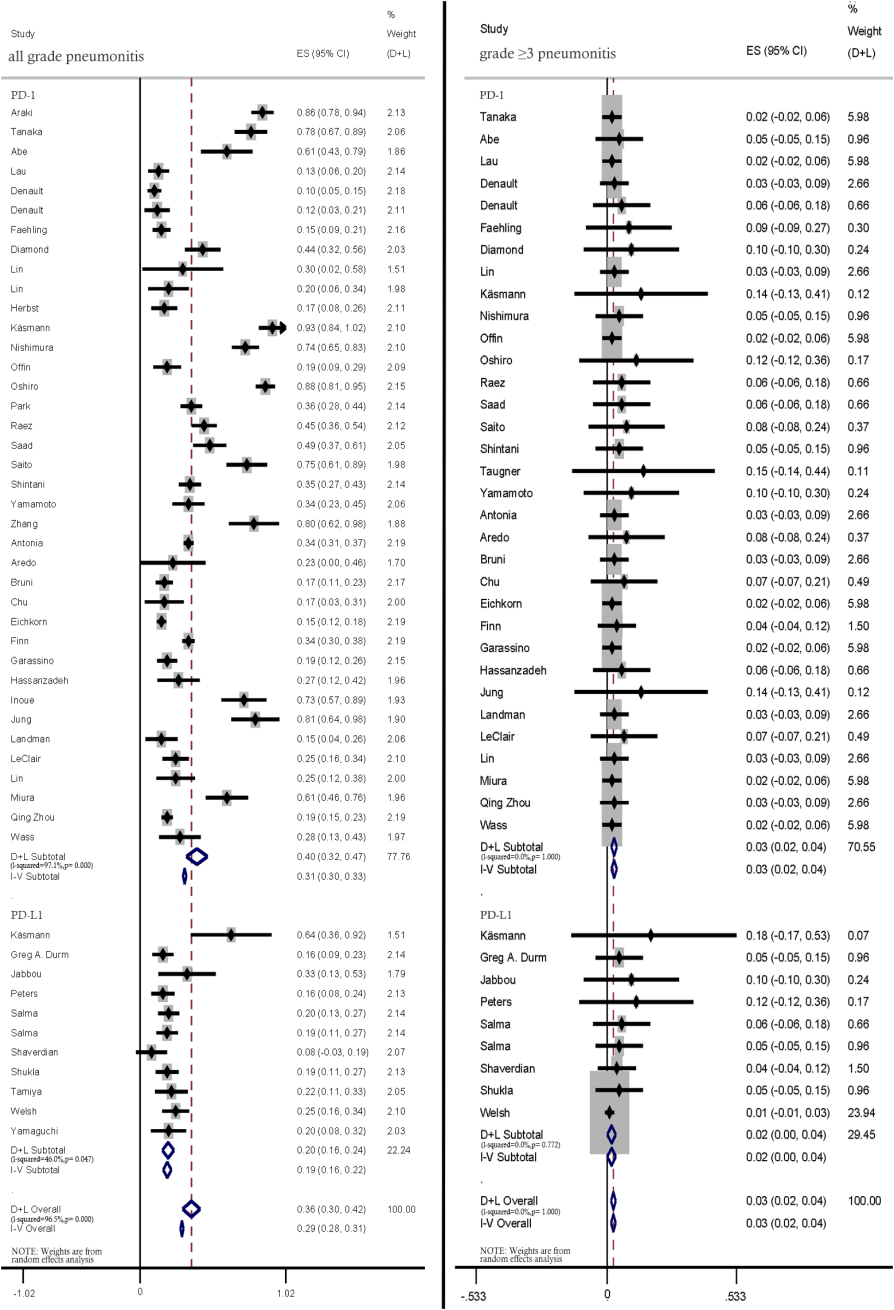
Subgroup analysis of all-grade and ≥ grade 3 pneumonitis incidence rates among patients treated with anti-PD-1 and anti-PD-L1 therapies.

Anti-PD1 therapy was associated with higher rates of all-grade pneumonitis than PD-L1 therapy (40%; 95% CI: 32%–47% vs. 20.0%; 95% CI: 16%–24%). Similar incidence rates of ≥ grade 3 pneumonitis were reported in all included studies (**Figure 6**).

#### 3.2.6 Asian patients and non-Asian patients

**Figure 7:**
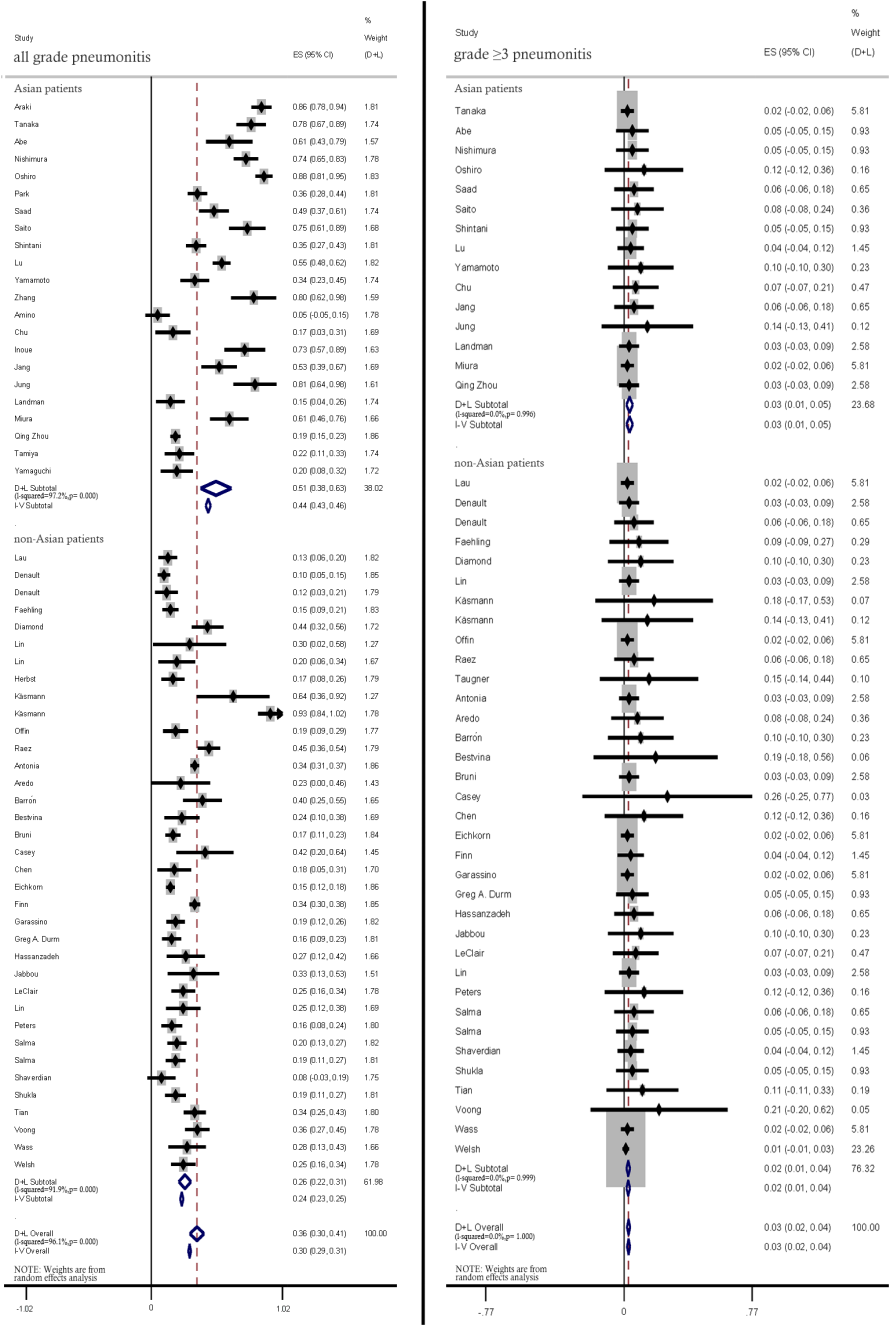
Subgroup analysis of all-grade and ≥ grade 3 pneumonitis incidence rates between Asian patients and non-Asian patients.

The pooled incidence rates of all-grade pneumonitis in Asian patients were significantly higher than those in non-Asian patients (51%; 95% CI: 38%–63% vs. 26%; 95% CI: 22%–31%). The incidence rates of ≥ grade 3 pneumonitis were not significantly different between Asian and non-Asian patients (3%; 95% CI: 1%–5% vs. 2%; 95% CI: 1%–4%) (**Figure 7**).

## 4 Discussion

The immunomodulatory properties of RT enable the development of rational treatment combinations with immunotherapy to achieve maximum local tumor control and inhibit systemic metastases. Previous studies have demonstrated that RT promotes lymphocytic infiltration, transforming “immunologically cold” tumors into “immunologically hot” tumors and establishing a tumor microenvironment (TME) that can respond to various ICIs. However, a consensus has not been achieved for the optimal dose and fraction scheme that can induce a change in the TME (10). Sustained damage induced by RT can lead to stress-induced aging in lung parenchymal stem cells, impairing their ability to repair tissue damage and enhancing inflammatory response through the secretion of cytokines and growth factors (65). Both RT and immunotherapy exert adverse effects on lung tissue. RT-induced cell damage and immunotherapy-induced immunological enhancement have common endpoints, namely the release of several cytokines/chemokines and the induction of damage-related signaling pathways. Therefore, this study systematically evaluated the incidence rates of all-grade and ≥ grade 3 pneumonitis in patients with LC who were treated with the combination of CRT and ICIs by summarizing available real-world data and clinical trials.

The incidence rates of pneumonitis in patients with LC after treatment with CRT-ICI combination reported in this study were slightly lower than those reported by Cui et al. (66). Cui et al. reported that the overall incidence of pneumonitis in patients receiving CRT-ICI combination therapy was 36.3%. However, Cui et al. did not provide statistics on the incidence of ≥ grade 3 pneumonitis and did not perform subgroup analyses. The number of studies included in previous meta-analyses was lower than that included in this study. Additionally, the data in previous meta-analyses were obtained from retrospective studies and a small number of prospective clinical trials. Furthermore, most of these studies have limited reliability with data obtained from single centers or limited sample size (66, 67).

This study compared the incidence rates of pneumonitis after treatment with CRT-immunotherapy combination in Asian and non-Asian patients with LC. The pooled incidence rates of all-grade pneumonitis in Asian patients were significantly higher than those in non-Asian patients (51%; 95% CI: 38%–63% vs. 26%; 95% CI: 22%–31%). However, the incidence rates of ≥ grade 3 pneumonitis were not significantly different between Asian and non-Asian patients (3%; 95% CI: 1%–5% vs. 2%; 95% CI: 1%–4%). The differential incidence rates of all-grade pneumonitis can be attributed to different genetic factors, such as single nucleotide polymorphisms of the TGF-β1-encoding gene in non-Asian and Asian populations. TGF-β1 is reported to be involved in the development of radiation lung injury. Additionally, TGF-β1 can trigger different responses depending on the environment and genetic makeup of the target cells. The plasma levels of TGF-β1 are upregulated during RT in patients who developed RP. In a study conducted by Yuan et al., which included 164 patients diagnosed with non-small cell lung cancer (NSCLC) undergoing radical chemoradiotherapy, it was observed that the CT/CC genotype of the rs1982073:T869C polymorphism was associated with a significantly reduced risk of developing radiation pneumonitis (RP) compared to the TT genotype. The hazard ratios were calculated to be 0.489 for grade ≥2 RP (P=0.013) and 0.390 for grade ≥3 RP (P=0.007), The polymorphisms rs1800469:C-509T and rs1800471:G915C showed weaker associations with the risk of radiation pneumonitis (RP). Nonetheless, rs1800469:C-509T exhibited a statistically significant association with grade ≥2 RP, with a hazard ratio (HR) of 0.568 (P=0.017). A study by Yuan et al. involving 164 NSCLC patients who received radical chemoradiotherapy showed that the rs1982073:T869C: CT/CC genotype was significantly associated with a lower risk of radiation pneumonitis (RP) compared to the TT genotype (HR = 0.489 for ≥ Grade 2 RP, P = 0.013; HR = 0.390 for ≥ Grade 3 RP, P = 0.007). The rs1800469:C-509T and rs1800471:G915C genotypes had weaker associations with RP risk, but the association between rs1800469:C-509T and ≥ Grade 2 RP was statistically significant (HR = 0.568, P=0.017). Furthermore, the distribution of the TGF-β1 rs1982073:T869C genotype exhibits variation between Asian and non-Asian populations, with the TT genotype occurring at frequencies of 29.8% and 31.9%, the TC genotype at 46.6% and 57.1%, and the CC genotype at 23.6% and 11.0%, respectively. Similarly, the TGF-β1 rs1800469:C-509T genotype also differs between these populations, with the TT genotype present at frequencies of 21.8% in Asians compared to 3.1% in non-Asians, the TC genotype at 48.6% versus 35.6%, and the CC genotype at 24.6% versus 61.3% (68–72). In the subgroup analysis of the PACIFIC study, patients with EGFR mutations demonstrated an increased incidence of pneumonia relative to those with wild-type EGFR (11.0% versus 3.8%),the frequency of *EGFR* mutations in Asian patients is higher than that in non-Asian patients (38.4% vs. 14.1%). Nonetheless, the proposition that EGFR mutations constitute a risk factor for pneumonia remains unverified in the absence of direct clinical investigations. Therefore, further clinical research is imperative to substantiate this potential association (73).

The discrepancies in the use of ICIs and combined treatment regimens between Asian and non-Asian studies may at least partially account for the observed outcomes. The consolidation use of durvalumab following concurrent CRT is a common protocol in studies involving the Asian population. However, in studies involving the non-Asian population, the frequent employment of PD-1 inhibitors (such as pembrolizumab or nivolumab) in conjunction with synchronous CRT and ICIs may lead to an increased risk of pulmonary toxicity. Thus, the incidence of ≥ grade 2 pneumonitis in non-Asian patients is comparable to that observed in Asian patients. For patients with multiple risk factors for pneumonitis (such as pre-existing pulmonary diseases, age > 75 years, and carriers of susceptibility genes), the use of pembrolizumab monotherapy can mitigate the risk of pneumonitis.

The incidence rates of all-grade and ≥ grade 3 pneumonitis in patients undergoing sequential CRT were higher than those in patients undergoing concurrent CRT in this study. This is in contrast to the findings of previous research, although the rates were still acceptable (73). Recent studies (56) have revealed that among patients with unresectable stage III NSCLC, approximately 30% exhibit disease progression after synchronous CRT, rendering them ineligible for subsequent immunotherapeutic interventions. The early initiation of immunotherapy can potentially provide immunotherapeutic benefits for all individuals undergoing synchronous CRT. Mechanistically, CRT modulates the immune microenvironment. Thus, the introduction of immunotherapy concurrently with CRT may exert synergistic therapeutic effects. This study suggests that concurrent CRT is a viable option for patients concerned about pneumonia-related complications. Further studies are needed to refine treatment protocols and minimize toxicity.

The Incidence rate of pneumonia in patients treated with anti-PD-1 agents were higher than those in patients treated with anti-PD-L1 agents, which is consistent with the findings of previous studies. As anti-PD-1 agents have two ligands (PD-L1 and PD-L2), PD-L1 inhibitors are associated with less toxicity. PD-L2 maintains physiological immune homeostasis. Hence, PD-L1 inhibitors specifically block the binding of PD-L1 and PD-1, preserving the function of macrophage PD-L2 (74). However, PD-L1 and PD-L2 have different binding partners, except for PD-1. PD-L2 and PD-L1 bind to RGMb and B7-1, respectively (74, 75). However, the incidence rates of pneumonitis were comparable between patients administered with anti-PD-1 and those administered with anti-PD-L1 agents, suggesting that the ability of anti-PD-1 agents to induce ≥ grade 3 pneumonitis is not higher than that of anti-PD-L1 agents (67). Thus, we hypothesized that the selection of anti-PD-1 and anti-PD-L1 therapies may not influence the incidence of pneumonitis associated with the RT-immunotherapy treatment combination.

This study analyzed the incidence of pneumonitis among patients undergoing conventional RT and SBRT. Compared with those in patients treated with SBRT, the incidence rates of all-grade (37%; 95% CI: 31%–42% vs. 26.0%; 95% CI: 20%–33%) and ≥ grade 3 pneumonitis (3%; 95% CI: 2%–4% vs. 1%; 95% CI: −1%–3%) were lower in patients treated with conventional RT. Advances in RT technology can contribute to improving the efficacy of the RT-ICI therapy combination. The shift from two-dimensional RT to three-dimensional RT techniques, including intensity-modulated RT and three-dimensional conformal RT, enables the implementation of RT with precision and decreases the exposure of adjacent critical structures to radiation (62). Intensity-modulated RT has similar outcomes as 3-dimensional conformal RT but is associated with a lower risk of RP (3.5% vs. 7.9%) (76). The advantages of SBRT are a high fractional dose and a short course of treatment. This allows the concentration of maximum radiation dose in the tumor target area with the surrounding healthy tissues receiving low radiation dose, which is beneficial for protecting the surrounding healthy tissue. Previous studies have reported that the immune activation effect of traditional RT may not be ideal. Standard doses of RT have limited effects on interferon release and may lead to the depletion of effector T cells in the TME. The adjustment of dosage and segmentation may optimize radiation-induced immunogenic cell death. During multisite RT, the high-dose irradiation of the target lesion using SBRT results in low-dose scattering of tumors outside the target area, improving the effectiveness of immunotherapy (77–79). Zhou et al. (80) conducted a study involving 29 untreated patients with PD-L1-positive NSCLC who received a combination of low-dose radiation (2 Gy per fraction), SBRT (30 Gy in 3 fractions), and sintilimab. The overall tolerability of the treatment was good. The incidence rate of ≥ grade 3 pneumonitis was 20.7% with 7 patients presenting grade 2–3 pneumonitis. The objective response rate and median progression-free survival were 57.1% and 8.6 months, respectively. The authors suggest that combining low-dose radiation with SBRT is a viable therapeutic strategy for advanced NSCLC. However, this evidence is preliminary and warrants further investigation. In cases where both traditional RT and SBRT are viable therapeutic options for patients with advanced NSCLC undergoing treatment with RT-immunotherapy combination, SBRT can be the preferred choice.

The timing of immunotherapy initiation is critical in patients with RP. In the PACIFIC trial (57), patients who developed grade 1 RP within 42 days of synchronous RT continued to receive maintenance therapy with durvalumab as planned. Thus, 35 patients with baseline grade 1 RP were treated with durvalumab and followed up for at least 1 year. The PACIFIC trial suggested that immunotherapy may be a feasible therapeutic option for patients with grade 1 RP. However, the PACIFIC trial did not report detailed clinical outcomes in patients with baseline grade 1 RP who were treated with duvalizumab. Takeya Sugimoto et al. (81) evaluated the safety and efficacy of duvalizumab in patients with baseline grade 1 radiation pneumonia. The study included patients with unresectable stage III NSCLC who presented with grade 1 RP within 42 days of concurrent CRT and received duvalizumab maintenance therapy. The study reported that 11 (31%) patients developed grade ≥2 pneumonia during treatment, 10 (28%) patients developed grade 2 pneumonia, and 1 (3%) patient developed grade 5 pneumonia. The median time from the initiation of duvalizumab to the onset of pneumonia was 2.8 months. Among the 10 patients who developed grade 2 pneumonia, 8 exhibited improvement after treatment with glucocorticoids and discontinuation of duvalizumab, 2 exhibited improvement only after the discontinuation of duvalizumab, and 6 received duvalizumab again without recurrence or exacerbation of pneumonia. The incidence of grade 2 pneumonia reported by Takeya Sugimoto et al. was slightly higher than that reported in the PACIFIC trial. Although the study was conducted with patients at high risk for pneumonia, the incidence of severe pneumonia was not high. Therefore, the study suggested that duvalizumab can be safely used for treating baseline grade 1 RP. These two studies are important for future advances in immunotherapy as they suggest that immunotherapy may be a feasible therapeutic regimen for patients with grade 1 RP. Additionally, these two studies provide initial evidence for the safety of ICIs in patients at high risk for developing pneumonia, such as those with grade 2 RP. Further clinical studies are needed to verify whether patients with grade 2 RP can be treated with ICIs.

This study has several limitations. As most included studies were associated with high heterogeneity, the random-effects model was used. To explore the sources of heterogeneity, subgroup analyses were performed. Meta-regression analysis revealed that the heterogeneity was attributed to study design, region, RT mode, combination therapy method, and ICI type. The significant discrepancy in pneumonia incidence rates observed between the studies conducted by Käsmann L and Amino Y, reported at 93% and 5% respectively, may be attributed to several potential factors: The prospective study design utilized by Käsmann L et al. incorporated systematic surveillance of pneumonia via regular imaging follow-ups, thereby likely enhancing the detection of subclinical pneumonia. Conversely, the retrospective approach adopted by Amino Y et al. may have contributed to the underreporting or underestimation of mild pneumonia cases.The timing of immune therapy varied notably: Käsmann L et al. started it right after concurrent chemoradiotherapy, whereas Amino Y et al. delayed until tumor recurrence, with a median initiation time of 9.3 months. Käsmann L Käsmann L et al. studied large-volume Stage III tumors with a median planning target volume (PTV) of 676 ccm, leading to significant radiotherapy coverage and higher lung dose parameters, including a V20 of 24.1% and a mean lung dose (MLD) of 13.9 Gy. In contrast, Amino Y et al.’s study, which lacked specific radiotherapy details, involved post-recurrence treatment, implying smaller target volumes or better lung function in their patients. In the study conducted by Käsmann L et al., which concentrated on large-volume Stage III tumors with a median planning target volume (PTV) of 676 ccm, the radiotherapy coverage was comprehensive, leading to elevated lung dose parameters, including a V20 of 24.1% and a mean lung dose (MLD) of 13.9 Gy. Conversely, the study by Amino Y et al. did not provide specific details regarding the radiotherapy parameters. Nevertheless, given that the treatment was administered following recurrence, it is plausible that the target volumes were reduced or the patients exhibited superior baseline lung function. Furthermore, 95% of the patients in the Amino Y study had a performance status (PS) of 0-1, suggesting good overall health and enhanced tolerability to treatment. The disparities previously mentioned may collectively explain the substantial differences in pneumonia incidence observed between the two studies.

Additionally, some unknown sources of heterogeneity may have not been included in the analysis. As the individual patient-level data were unavailable, statistical efficacy was not sufficient to distinguish the effects of various factors on the occurrence of pneumonitis.

## 5 Conclusions

This comprehensive meta-analysis of 4226 patients with LC systematically and quantitatively explored the safety of the combination of ICI and RT/CRT. The combination of RT/CRT and ICIs did not significantly increase the incidence of ≥ grade 3 pneumonitis. The safety of different ICIs, timings of immunotherapy, and RT modes and different populations was demonstrated. These findings may help clinicians in designing treatment plans for patients with LC.

## Data Availability

The datasets presented in this study can be found in online repositories. The names of the repository/repositories and accession number(s) can be found in the article/supplementary material.
